# Antioxidant Effects of *Schisandra chinensis* Fruits and Their Active Constituents

**DOI:** 10.3390/antiox10040620

**Published:** 2021-04-18

**Authors:** Dalia M. Kopustinskiene, Jurga Bernatoniene

**Affiliations:** 1 Institute of Pharmaceutical Technologies, Faculty of Pharmacy, Medical Academy, Lithuanian University of Health Sciences, Sukileliu pr. 13, LT-50161 Kaunas, Lithuania; DaliaMarija.Kopustinskiene@lsmuni.lt; 2 Department of Drug Technology and Social Pharmacy, Faculty of Pharmacy, Medical Academy, Lithuanian University of Health Sciences, Sukileliu pr. 13, LT-50161 Kaunas, Lithuania

**Keywords:** *Schisandra chinensis*, lignan, schisandrin B, antioxidant, pro-oxidant, mitochondria

## Abstract

*Schisandra chinensis* Turcz. (Baill.) fruits, their extracts, and bioactive compounds are used in alternative medicine as adaptogens and ergogens protecting against numerous neurological, cardiovascular, gastrointestinal, liver, and skin disorders. *S. chinensis* fruit extracts and their active compounds are potent antioxidants and mitoprotectors exerting anti-inflammatory, antiviral, anticancer, and anti-aging effects. *S. chinensis* polyphenolic compounds—flavonoids, phenolic acids and the major constituents dibenzocyclooctadiene lignans are responsible for the *S. chinensis* antioxidant activities. This review will focus on the direct and indirect antioxidant effects of *S. chinensis* fruit extract and its bioactive compounds in the cells during normal and pathological conditions.

## 1. Introduction

*Schisandra chinensis* Turcz. (Baill.) belongs to the Schisandraceae family. The plants are native to northeastern China, Japan, Korea, Manchuria, and the Far East part of Russia. Their purple-red berries are called five-flavor fruits because of the sweet, bitter, pungent, salty, and sour taste [1,2,3,4,5]. *S. chinensis* is widely used as an herbal supplement in traditional Chinese medicine and in Western phytotherapy [1,2,4,5], whereas in Russia—as a potent adaptogen, improving disease and stress tolerance, and increasing energy, endurance, and physical performance [2,3].

*S. chinensis* is used as a preservative and an additive in food technology to enhance the flavor, taste, and nutritional value to the food [2]. The dried fruits of the *S. chinensis* and their extracts are helpful in the treatment of neurological, cardiovascular, and gastrointestinal disorders, in decreasing fatigue, reducing obesity, and protecting from mitochondrial dysfunction, insomnia, and the excessive sweating [1,2,5]. They stimulate immunity, act as a tonic, and exert antioxidant, anti-inflammatory, antiviral, anticancer, anti-aging, anti-diabetic, and liver- and skin-protecting activities [1,2,3,4,5].

## 2. Chemical Properties of *Schisandra chinensis* Fruit Constituents

*S. chinensis* fruits contain about 1.5% sugars (polysaccharides and monosaccharides; glucose, fructose, galactose, and arabinose), tannins (hydrolysable, e.g., gallic acid esters, and condensed, e.g., proanthocyanidins and catechol-type tannins), color substances (mainly anthocyanins), and about 3% essential oils, with sesquiterpenes as the dominant compounds [6]. α-Bergamotene, β-chamigrene, β-himachalene, and ylangene are the main components of essential oils (about 75%), whereas oxygenated sesquiterpenes, monoterpenes, and oxygenated monoterpenes comprise the smaller part (about 5%) [6]. Chemical investigations also revealed the presence of triterpenoids (lanostane and cycloartane-type triterpenoids and nortiterpenoids), organic acids (citric, fumaric, malic, and tartaric acids), phenolic acids (chlorogenic, gentisic, p-hydroxybenzoic, p-coumaric, protocatechuic, syringic, and salicylic acids) [7,8], flavonoids (quercetin, isoquercitrin, rutin, and hyperoside) [8], vitamins C and E, phytosterols, and bioelements (Cr, Cu, Co, Ca, Mg, Fe, Zn, Mn, B, Ni,) [1,7,9].

**Figure 1:**
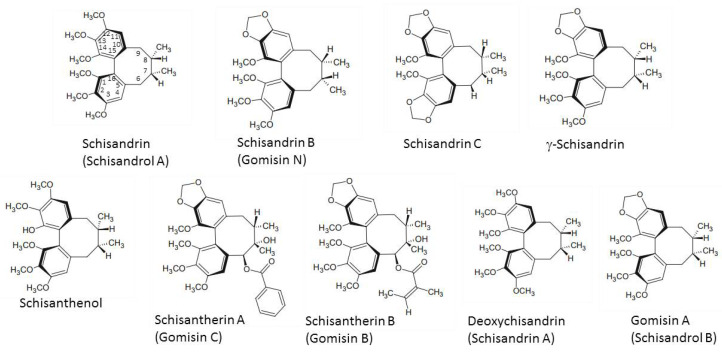
Chemical structures of main *Schisandra chinensis* lignans.

The major active compounds of *S. chinensis* (Figure 1) are dibenzocyclooctadiene lignans [1]. Schisandrin is the most dominant *S. chinensis* lignan found in the amounts of 2.2–5.3 mg/g in *S. chinensis* fruit [2,10].

Anticancer activity of *S. chinensis* lignans is decreased in the presence of a hydroxyl group at the C7 position, thus resulting in increased hydrophilicity and decreased permeability into the lipid bilayer [11], whereas a methylenedioxy group between C12 and C13 enhanced anticancer activity. A 1,2,3-trimethoxy moiety, a 6-acyloxy group, and the absence of a 7-hydroxy group resulted in P-glycoprotein inhibition and also increased *S. chinensis* anticancer efficacy [12]. Gomisin N and deoxyschisandrin were the most effective anticancer lignans of *S. chinensis* [11,12]. *S. chinensis* lignans without an ester group at C6, a hydroxyl group at C7, or a methylene dioxy moiety, and with an R-biphenyl configuration possess strong antiplatelet activity, with 6,7-dehydroschisandrol A as the most active compound [13]. The exocyclic methylene group in *S. chinensis* lignan structure is necessary for the antioxidant activity, which is enhanced even more in the presence of the benzoyloxy group [14].

## 3. Bioavailability of *Schisandra chinensis* Fruit Extract and Its Constituents

The bioavailability studies of *S. chinensis* products were mainly performed in animals. A maximum concentration of schisandrin of 0.08 ± 0.07 and 0.15 ± 0.09 μg/mL was achieved after oral administration of 3 g/kg and 10 g/kg of *S. chinensis* fruit extract in rats [15]. In a parallel study, schisandrin (10 mg/kg, administered intravenously (i.v.) or orally (p.o.)) and the herbal extract of *S. chinensis* (3 g/kg and 10 g/kg, p.o.) were given indivi-dually to rats [15]. The dose of *S. chinensis* (3 g/kg) was equivalent to schisandrin (5.2 mg/kg), whereas the dose of *S. chinensis* (10 g/kg) was equivalent to schisandrin (17.3 mg/kg) [15]. Thus, in rats, the oral bioavailability of schisandrin was approximately 15.56 ± 10.47% [15]. When Sprague-Dawley rats were administered 2 mg/kg (i.v.) or 10 mg/kg (intragastrically (i.g.)) of schisandrol B, or 6 mL/kg (i.g.) of *S. chinensis* extract (equivalent to 15 mg/kg schisandrol B), the oral absolute bioavailability of schisandrol B was approximately 18.73% and 68.12%, respectively [16]. Schisandrin B could modulate cytochrome P450 3A activity (CYP3A) in vivo in rats and also altered the pharmacokinetic profiles of other CYP3A substrates [17]. The tissue distribution studies showed that schisandrin B [18] and schisandrol B were distributed throughout several tested tissues and accumulated mainly in in the liver and kidneys [16,18]. Absolute oral bioavailability of schisandrin B depended on the sex of animals—it was approximately 55.0% for female rats and 19.3% for male rats [19]. The linear pharmacokinetics properties were observed within the range of the tested oral dose (10, 20, and 40 mg/kg rat) of schisandrin B [19]. Schisandrin B was extensively distributed in ovary and adipose tissue [19]. The urinary, biliary, and fecal excretion of schisandrin B was very low; schisandrin B was excreted mainly in the form of metabolites [20].

In alternative medicine, *S. chinensis* dried fruit powder is usually administered to patients at a dose of 0.5–1.5 g twice per day before meals over a period of 20–30 days [3]. No serious adverse effects were reported during use of *S. chinensis*; however, overdose may cause dyspnea, restlessness, or insomnia [2,3,5].

## 4. Biological Activity of *Schisandra chinensis* Fruit Extract and Its Constituents: Main Mechanisms of Action

**Figure 2:**
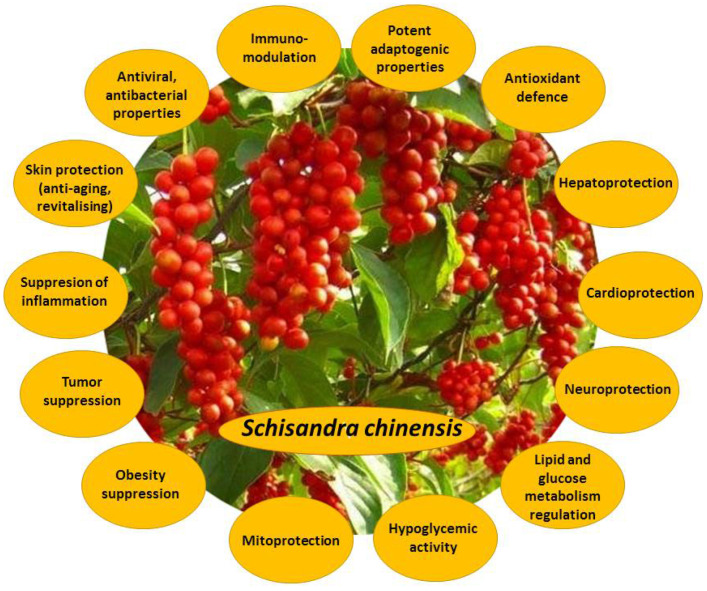
Main biological effects of *Schisandra chinensis* fruit extracts and their bioactive compounds.

Dried *S. chinensis* fruits, their extracts, and their bioactive constituents exert a wide variety of beneficial effects under normal and pathological conditions (Figure 2). *S. chinensis* bioactive compounds are antioxidants, detoxifiers, powerful hepatoprotectors, hypoglycemic agents, inflammation suppressors, neuro- and cardioprotectors, immunostimulants, and tumor suppressors [1,2,3,5]. They demonstrate antibacterial and antiviral properties, and suppress platelet aggregation; they also are potent adaptogens and ergogens, capable to decrease fatigue and support the normal functioning of cellular powerhouses—mitochondria [2,7]. *S. chinensis* bioactive compounds are also potent skin-protectors. Their anti-aging and revitalizing actions comprise moisturizing, toning, irritation-soothing, and wound-healing, reducing dilatation of blood vessels and restoring the skin protective barrier.

Principal mechanisms of beneficial actions of *S. chinensis* bioactive compounds include activation of the antioxidant defense system, reducing the levels of aspartate aminotransferase, alanine aminotransferase, and serum and liver glutamic pyruvic transaminase, as well as inactivation of cytochrome P450 [21]. *S. chinensis* bioactive compounds inhibit pro-oxidant signaling pathways: cyclooxygenase 1 and 2 (COX-1 and 2) [22], nitric oxide production [23], and gene expression of pro-inflammatory cytokines [24]. Furthermore, *S. chinensis* constituents block calcium channels (Ca^2+^) [25] and inhibit the opening of the mitochondrial permeability transition pore (mPTP), thus protecting from cell death [25,26,27]. In tumor cells, *S. chinensis* bioactive compounds can reverse multidrug resistance dependent on P-glycoprotein (Pgp-MDR) [28] and sensitize tumor cells to antitumor treatments, e.g., with doxorubicin [29,30]. They can also promote cell cycle arrest, thus suppressing proliferation and activating apoptosis and autophagy [31,32,33].

## 5. Antioxidant Activity of *Schisandra chinensis* Fruit Extract and Its Constituents

Impaired balance in pro-oxidant and antioxidant homeostasis causes oxidative stress, which enhances the production of toxic reactive oxygen species (ROS). ROS in the cells are mainly generated in mitochondria as by-products of the mitochondrial respiratory chain, whereas some of them can be derived also from redox metal ion-related and enzymatic sources [34,35]. ROS are neutralized by enzymatic and non-enzymatic endogenous antioxidant defense systems. Superoxide dismutase (SOD), catalase (CAT), glutathione peroxidase (GPx), and glutathione reductase (GR) belong to the enzymatic defense system, while small antioxidant molecules, such as vitamin C, vitamin E, and reduced glutathione (GSH), make part of non-enzymatic defense [34].

**Figure 3:**
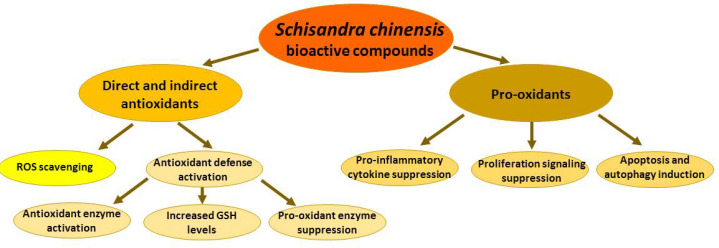
Antioxidant and pro-oxidant activities of *Schisandra chinensis* fruit extracts and their bioactive compounds. ROS—reactive oxygen species.

As with many plant-derived antioxidants, *S. chinensis* active compounds can directly scavenge reactive oxygen species, activate the antioxidant defense system under normal conditions, and act as pro-oxidants under pathological conditions (Figure 3).

Regarding *S. chinensis* polyphenolic compounds: Flavonoids, phenolic acids, and the major constituents of dibenzocyclooctadiene lignans are considered to be responsible for the antioxidant activities of *S. chinensis* fruit extract [2,4].

### 5.1. Direct ROS Scavenging by Schisandra chinensis Fruit Extract and Its Constituents

The direct ROS scavenging capability of *S. chinensis* ethanolic extracts was demonstrated by Mocan A et al. using the DPPH bleaching assay, Trolox equivalent antioxidant capacity assay, hemoglobin ascorbate peroxidase activity inhibition, and the inhibition of lipid peroxidation catalyzed by cytochrome *c* assays and an electron paramagnetic resonance spectroscopy [8]. *S. chinensis* fruit ethanolic extract could directly scavenge ROS, thus alleviating hydrogen peroxide (H_2_O_2_)-induced inhibition of C2C12 cell growth [36]. Moreover, different schisandrins could neutralize ROS from human polymorphonuclear leukocytes stimulated with phorbol myristate acetate [37].

The relative order of the strength of ROS scavenging of schisandrins depended on their conformation and the presence of the dioxymethyl group capable of attracting electrons, thus facilitating radical attack: S(-)-schisandrin B > S(+)-schisandrin > schisandrin C > schisandrin B [37]. The ROS scavenging effect of schisandrin B was similar to that of vitamin C [38]. Fenton reaction, xanthine-xanthine oxidase, or UV-irradiation of riboflavin assays revealed that the *S. chinensis* lignan schisanhenol could neutralize ROS better than vitamin E in an experimental model of tetradecanoylphorbol acetate-stimulated human neutrophils [39]. Furthermore, in in vitro studies, *S. chinensis* fruit aqueous extract protected human blood lymphocyte DNA from oxidant challenge by H_2_O_2_, evaluated by comet assay [40].

### 5.2. Effects of Schisandra chinensis Fruit Extract and Its Constituents on Enzymatic and Non-Enzymatic Endogenous Antioxidant Defense Systems

*S. chinensis* bioactive compounds exerted antioxidant activities in many tissues including the brain [41]. In a D-galactose-induced Wistar rat neurotoxicity model, *S. chinensis* aqueous or ethanolic extracts decreased SOD, CAT, and total antioxidants, and maintained the normal levels of GSH, malondialdehyde (MDA), and nitric oxide (NO) in the serum, striatum, hippocampus, and prefrontal cortex, thus ameliorating cognitive deficits assessed using the Morris water maze and the step-down type passive avoidance test [42]. Schisandrin B (10, 25, or 50 mg/kg administered orally (p.o.) for 7 days) could increase the levels of antioxidant enzymes, such as SOD, GPx, and cellular GSH in mice, and suppress lipid peroxidation in scopolamine- and cisplatin-induced cerebral oxidative stress [43]. Furthermore, schisandrin B exerted neuroprotective activity by reducing MDA levels and ROS generation, while in the meantime enhancing SOD activity and GSH production in the mice force swimming stress model, thus reducing anxiety-like behavior [44]. Schisandrin B (5, 10, or 20 μM) could suppress the production of ROS in microglia-neuron co-cultures [45]. Antioxidant effects of the *S. chinensis* lignan deoxyschisandrin (4, 12, and 36 mg/kg *i.g.* for 14 days) were investigated on the amyloid-beta (1-42) Aβ(1-42)-induced memory impairment model in mice [46]. Deoxyschisandrin improved Aβ(1-42)-induced short-term and spatial memory impairments assessed using the Y-maze and water maze tests. In the cerebral cortex and hippocampus of mice, deoxyschisandrin restored the suppression of SOD and GPx activities, increased GSH levels and the GSH/oxidized glutathione (GSSG) ratio, and decreased MDA and GSSG levels [46], thus alleviating cognitive decline in Alzheimer’s disease [46]. The effects of *S. chinensis* lignan schisandrin C (15 μg/kg or 150 μg/kg/day for five days in the lateral cerebral ventricles using sterotaxically implanted cannula) on pathological changes and memory impairment were evaluated in the Aβ(1-42)-induced Alzheimer’s disease model in mice [47]. Schisandrin C restored cognitive functions and decreased neuronal injury by inhibiting total cholinesterase, enhancing SOD and GPx activities and increasing GSH levels in the hippocampus and cerebral cortex [47]. In the scopolamine-treated mice model, schisanthenol (10, 30, 100 mg/kg/day i.p. for seven days) improved learning and memory ability assessed by the Morris water maze test [48]. In mice, hippocampus schisanthenol enhanced the activity of SOD and GPx, while it decreased the content of MDA and acetylcholinesterase [48].

*S. chinensis* bioactive compounds were hepatoprotective in various liver intoxication models [1,2]. In in vitro studies, *S. chinensis* lignans schisanthenol, schisandrin B, and schisandrin C at a concentration of 1 mM suppressed iron/cysteine induced lipid peroxidation, assessed by a decrease in MDA formation in rat liver microsomes, and did so more effectively than vitamin E [49]. In AML12 hepatocytes, schisandrin B (15 μM) could induce glutathione antioxidant response [50]. *S. chinensis* fruit extract and its active compound—schisandrin B—protected against carbon tetrachloride-induced hepatotoxicity by increasing mitochondrial GSH levels and enhancing activities of GR, GPx, and glutathione S-transferases in carbon tetrachloride-intoxicated mice [51,52,53]. Schisandrin B (3 mmol/kg/day p.o. for three days) was also protective in the carbon tetrachloride-induced mice hepatotoxicity model by increasing the hepatic vitamin C and vitamin E levels, as well as mitochondrial GSH levels [54]. Lignan-enriched *S. chinensis* fruit extract ameliorated the hepatic antioxidant/detoxification system in rats after aflatoxin beta 1 or cadmium chloride challenge by increasing hepatic GSH levels and hepatic GR, and glutathione S-transferase activities [55]. In addition, 5-hydroxymethyl-2-furfural isolated from *S. chinensis* fruit (7.5, 15, and 30 mg/kg p.o. for seven days) was hepatoprotective in the acute alcohol-induced liver oxidative injury model in mice by decreasing the levels of MDA and increasing CAT, GPx, and SOD activities in liver tissue [56]. 

*S. chinensis* fruit extract and its active constituent schisandrin B exerted cardioprotective effects by enhancing the heart antioxidant defense system [57,58,59,60,61]. *S. chinensis* fruit extract protected from adriamycin-induced cardiotoxicity in rats by decreasing MDA levels and increasing activities of myocardial GPx and SOD [60]. Lignan-enriched *S. chinensis* extract protected heart from oxidative damage in an in vivo model of myocardial infarction and an ex vivo model of myocardial ischemia-reperfusion injury in rats [61]. Moreover, schisandrin B and C (10–30 μM), but not schisandrin A, stimulated the cytochrome P-450-catalysed NADPH oxidation reaction in in vitro studies of rat heart microsomes and/or ROS production in rat hearts (a single dose of 1.2 mmol/kg), resulting in the increase in mitochondrial GSH levels during, thus protecting against ischemia/reperfusion injury [59]. During doxorubicin-induced cardiomyopathy in mice, schisandrin B (25–100 mg/kg/day per os for five days) reduced lipid peroxidation, prevented nitrotyrosine formation, and suppressed metalloproteinase activation in the heart [58]. During myocardial ischemia/reperfusion (40 min + 1 h) in rats, schisandrin B (20 mg/kg) decreased MDA levels and increased total SOD activity, thus attenuating oxidative injury [57]. 

Schisandrin B in HK-2 cells (2.5–10 µM) and in mice (20 mg/kg/day per os for four weeks) decreased renal MDA levels and enhanced GSH production in cyclosporine A-induced nephrotoxicity [62]. Furthermore, in gentamicin-induced nephrotoxicity in rats, schisandrin B (1–10 mg/kg/day for 15 days) exerted nephroprotective effects by enhancing renal mitochondrial antioxidant status: increasing GSH and alpha-tocopherol levels and activating SOD [26]. 

*S. chinensis* ethanol extract and ethanol-water extract significantly decreased the pulmonary MDA levels and increased SOD activity and GSH levels in a guinea pig model of cough hypersensitivity induced by 14 days of cigarette smoke exposure [63].

### 5.3. Effects of Schisandra chinensis Fruit Extract and Its Constituents on the Pro-Oxidant Enzymes

Besides the mitochondrial electron transport chain, the potential ROS sources in the cells are enzymatic reactions catalyzed by the pro-oxidant enzymes: NADPH oxidases and the arachidonic acid-metabolizing enzymes—cyclooxygenases and lipoxygenases, xanthine oxidase, nitric oxide (NO) synthases, and the cytochrome P450 [35]. *S. chinensis* fruit extract and its bioactive compounds could effectively protect against inflammation due to the suppression of pro-oxidant enzyme activities [2,5].

*S. chinensis* fruit water extract inhibited gene expression of inducible NO synthase (iNOS) and cyclooxygenase-2 (COX-2) in lipopolysaccharide (LPS)-stimulated RAW 264.7 macrophage cells, thus suppressing the protein kinase B (Akt)-dependent nuclear factor-kappa B (NF-κB) signaling pathway without any cytotoxic effect [64]. The *S. chinensis* lignan schisandrin (5–100 µM) demonstrated anti-inflammatory activities in vitro by inhibiting NO production, iNOS, and COX-2 expression in the LPS-treated RAW 264.7 macrophage cell line, thus suppressing NF-κB, c-Jun N-terminal kinase (JNK), and the p38 mitogen-activated protein kinase (MAPK) signaling pathways [65]. Schisantherin A (0.5–25 mg/L), in a concentration-dependent manner, could block NF-κB and MAPK signaling in lipopolysaccharide (LPS)-stimulated RAW 264.7 macrophages by decreasing NO production, iNOS, and COX-2 activities [66]. Schisandrin A (9–47 μM) exerted anti-inflammatory effects in interleukin-1β-stimulated human osteoarthritis chondrocytes by reducing NO production, suppressing iNOS and COX-2 activities, thus blocking NF-κB and MAPK signaling [67]. Schisandrin B (5, 10 or 20 μM) protected against microglial-mediated inflammatory injury in microglia-neuron co-cultures by downregulating NADPH oxidase and other pro-inflammatory enzymes [45].

## 6. Effects of *Schisandra chinensis* Fruit Extract and Its Constituents on Mitochondria

**Figure 4:**
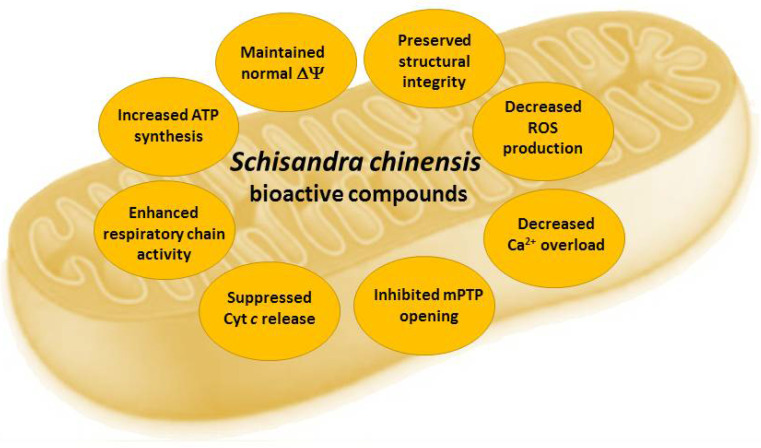
Effects of *Schisandra chinensis* fruit extracts and their bioactive compounds in mitochondria. ΔΨ—mitochondrial membrane potential, mPTP—mitochondrial permeability transition pore, ROS—reactive oxygen species.

Mitochondria play a crucial role in calcium homeostasis, apoptosis, and metabolism regulation under normal and pathological conditions in the cells [68]. Mitochondria are responsible for cellular energy supply via oxidative phosphorylation, generating ROS as a by-product during this process [69]. The excessive ROS trigger numerous events in signal transduction pathways modulating inflammation, apoptosis, proliferation, and immune response [68,69]. *S. chinensis* fruits, their extracts, and their bioactive constituents help to restore impaired mitochondrial functions, acting as mitoprotective agents (Figure 4).

In the Aβ(1-42) oligomer-treated rat primary hippocampal neuron model, schisandrin (2 μg/mL) alleviated impaired mitochondrial functions, energy metabolism, mitochondrial biogenesis, and dynamics [70]. Schisandrin restored mitochondrial membrane potential cytochrome c oxidase activity, protected the opening of mitochondrial permeability transition pore, and decreased the release of cytochrome c [70]. Furthermore, schisandrin improved ATP production, citrate synthase activity, and the process of mitochondrial fusion and fission [70]. The *S.chisandra* lignan gomisin J up-regulated apoptosis signaling and decreased the dissociation of hexokinase II from the voltage-dependent anion channel (VDAC) in mitochondria, thus reducing aerobic glycolysis in glioma cell lines [71]. Mixture of *S. chinensis* extract and ascorbic acid restored mitochondrial respiration, improved cognitive function, and induced synaptic plasticity in mice [72]. 

Schisandrin B protected from ROS generation, lipid peroxidation, protein oxidation, and DNA damage in tert-butyl hydroperoxide-injured human keratinocyte-derived HaCaT cells by reducing the loss of the mitochondrial membrane potential, restoring adenosine triphosphate levels, and enhancing the expression of key antioxidant enzymes—catalase, superoxide dismutase, glutathione peroxidase, and heme oxygenase-1 [73]. The *S. chinensis* lignan schisandrin A protected against H_2_O_2_-induced cytotoxicity and DNA damage in C2C12 cells by restoring ATP levels, maintaining the mitochondrial membrane potential and inhibiting cytochrome c release into the cytoplasm [74]. The *S. chinensis* lignan gomisin N increased the expression of mitochondria fatty acid oxidation and biogenesis genes in C2C12 myotubes [75]. Long-term schisandrin B treatment (1–30 mg/kg/d for 15 days) in cerebral ischemia/reperfusion (I/R) injury [27] and gentamicin-induced nephrotoxicity [26] models in rats revealed that schisandrin B enhanced cerebral mitochondrial antioxidant status, preserved mitochondrial structural integrity, reduced Ca^2+^ load and cytochrome c release, and protected from mPTP opening [26,27]. Schisandrin B protected against carbon tetrachloride-induced hepatic damage by decreasing the sensitivity of mice liver mitochondria to the Ca^2+^-stimulated permeability transition, suppressing the Ca^2+^-loading, ROS production, and cytochrome c release [76]. Schisandrin B and γ-schisandrin (2.5–5.0 µM) was protected from hypoxia/reoxygenation-induced apoptosis in H9c2 cardiomyocytes by decreasing the sensitivity to Ca^2+^-induced mPTP and increasing the mitochondrial membrane potential in both normal and damaged cells [25].

## 7. Conclusions and Future Perspectives

*S. chinensis* fruit extracts and their active compounds are potent antioxidants capable to scavenge ROS directly, activate the cellular antioxidant defense system components, and inhibit pro-oxidant enzymes, thus suppressing inflammation signal transduction pathways and protecting from apoptosis. *S. chinensis* bioactive compounds decrease the levels of liver function markers, block pro-oxidant enzyme activities, suppress inflammation, and exert anticancer effects, activating apoptosis and autophagy in cancer cells.

*S. chinensis* fruit preparations are well-tolerated and do not have serious adverse effects; therefore, they could be used as potential remedies to alleviate oxidative stress injuries and to restore normal cellular energy supply.

## Data Availability

Not applicable.
